# Comparison of the French and CARDS classifications for lumbar degenerative spondylolisthesis: reliability and validity

**DOI:** 10.1186/s12891-019-2753-3

**Published:** 2019-08-20

**Authors:** Chao Kong, Xiangyao Sun, Junzhe Ding, Machao Guo, Xiangyu Li, Shibao Lu

**Affiliations:** Department of Orthopedics, Beijing Xuanwu Hospital, Capital Medical University, Beijing, 100053 China

**Keywords:** Lumbar degenerative spondylolisthesis, CARDS classification, French classification, Reliability, Clinical outcome

## Abstract

**Background:**

The aim of this study was to compare the reliability and validity of the CARDS and French classification systems for lumbar DS.

**Methods:**

Between May 2013 and December 2016, 158 consecutive patients diagnosed with single-level lumbar DS were included in this study, and all underwent lumbar fusion. All patients underwent long-cassette standing anterioposterior and lateral radiographs of the spine preoperatively and postoperatively. The images were graded according to the CARDS and French classification systems by two orthopedic spinal surgeons and two orthopedic spinal fellows, independently. Clinical outcome measures used were the visual analog scale, Oswestry Disability Index, and the 36-Item Short Form Health Survey. Clinical data were collected before surgery and 1 year after surgery.

**Results:**

A total of 146 patients were finally included in this study and followed up for at least 1 year. When grading using the CARDS system, the κ values for inter- and intraobserver reliability were 0.837 and 0.869, respectively, representing perfect agreement. The interobserver κ value for the French classification was 0.693 and the intraobserver κ value was 0.743, both representing substantial agreement. CARDS Type D patients have higher preoperative back pain scores and better improvement after surgery compared with non-Type D patients. Mean back and leg pain was worse in French Type 5 patients, while the most significant improvement was also seen in Type 5 patients after surgery.

**Conclusions:**

Both CARDS and French classification systems have acceptable reliability and validity. The CARDS system is easier to utilize and has better reliability.

**Level of evidence:**

IV

## Background

Lumbar degenerative spondylolisthesis (DS) was first termed by Newman and Stone [1], noting the anterior migration of vertebrae without a pars defect. Recently, the North American Spine Society (NASS) defined lumbar DS as acquired anterior displacement of one vertebra over the subjacent vertebra, associated with degenerative changes, without an associated disruption of defect in the vertebral ring [2]. Although surgery is recommended for patients who are refractory to conservative treatment, the optimal surgical management remains controversial [3]. Lubelski et al. [4] conducted a large survey that evaluated surgical treatment patterns for lumbar DS among 445 US spinal surgeons, in which substantial variability was found, especially in patients without associated back pain. One possible reason for the variability of analysis or surgical treatments was the heterogeneous nature of lumbar DS [4].

Previously, lumbar DS was classified based on etiology and slip grade, which provides limited clinical utility in guiding surgical treatment since the magnitude of slip rarely exceeds 30% [5, 6]. What’s more, the Meyerding classification does not take other morphologic parameters such as segmental kyphosis or disc height into consideration, which are related to clinical outcomes. The variability of radiographic features suggests that lumbar DS is a heterogeneous disease and requires a specific grading system. In 2014, Kepler and his colleagues proposed The Clinical and Radiographic Degenerative Spondylolisthesis (CARDS) classification system of lumbar DS based on radiographic characteristics and clinical manifestations [7]. In the same year, the French classification for lumbar DS was developed and reported by the French Society for Spinal Surgery, which was based on the adult spinal deformity classification system developed by Schwab et al. [8]. Every new classification should be tested before being widely used in clinical assessment. However, no data or studies have compared the reliability and validity between the CARDS and French classifications. In this study, we retrospectively followed 158 patients with single-level lumbar DS, aiming to compare the reliability and validity between the two classifications.

## Method

### Patients demographics

Between May 2013 and December 2016, 158 consecutive patients diagnosed with single-level lumbar DS were included in this study; all of whom underwent fusion surgery. Twelve patients were excluded because of incomplete data, leaving 146 patients in total who were included and followed up for at least 1 year. Patients were excluded if they had a pars defect, hip disorders, previous spinal surgery or trauma, or incomplete data.

Patients data were recorded as age, sex, and body mass index (BMI). Clinical outcome measures used were the visual analog scale (VAS), Oswestry Disability Index (ODI), and the 36-Item Short Form Health Survey (SF-36). Clinical data were collected before surgery and 1 year after surgery by independent assessors.

### Radiological assessment

Radiographic measurements were made on long-cassette standing anteroposterior and lateral radiographs of the spine using Surgimap (New York, USA, version: 2.2.9.9.1). Parameters measured included intervertebral disc height (IDH), pelvic incidence (PI), pelvic tilt (PT), sagittal vertical axis (SVA), lumbar lordosis (LL), segmental lordosis (SL), and percentage of vertebral slippage.

**Fig. 1:**
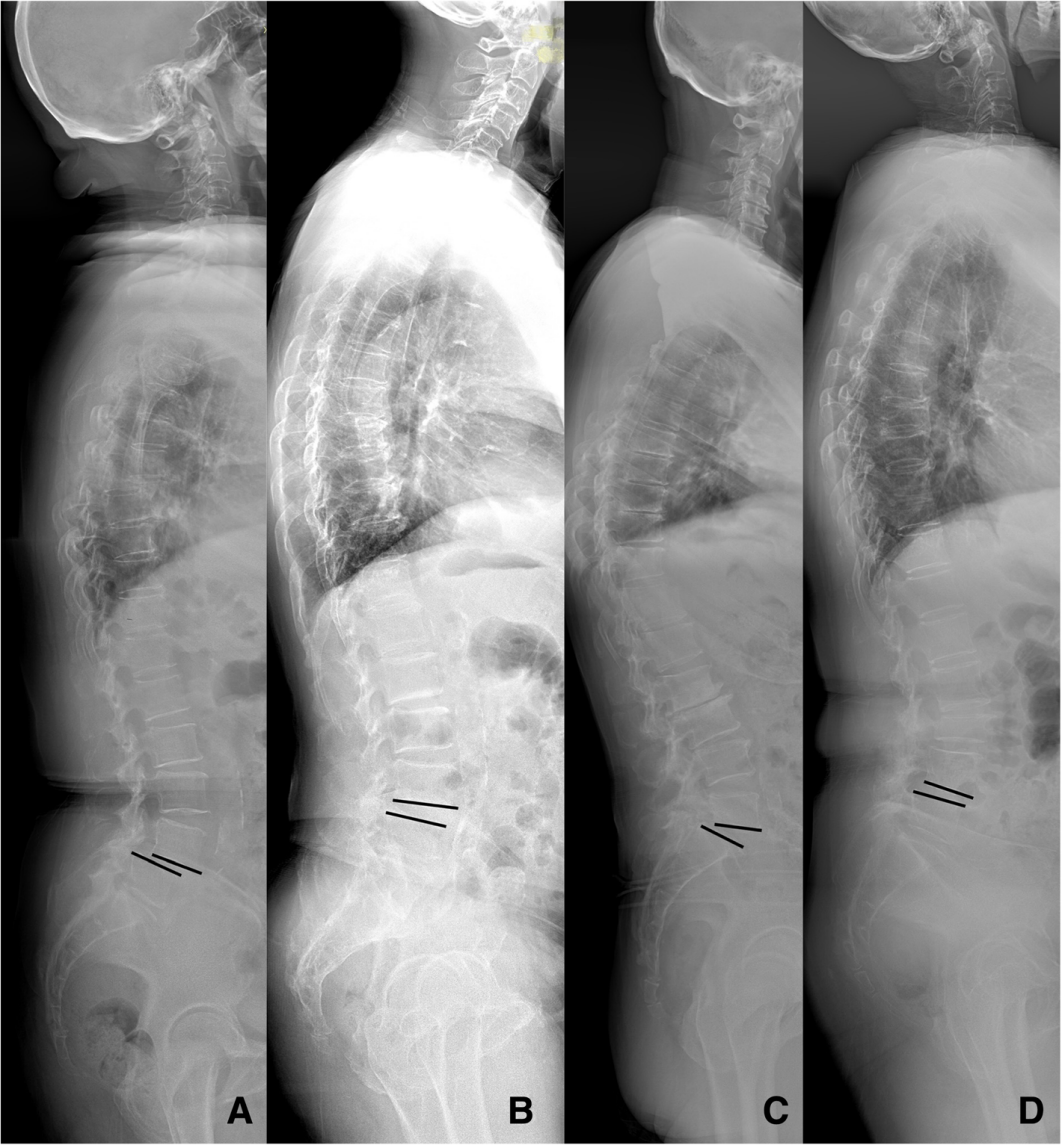
Diagram of the CARDS classification. **a** Type A, advanced disc space collapse at L4//5 without kyphosis; **b** Type B, disc height partially preserved with translation less than 5 mm; **c** Type C, disc height partially preserved with translation more than 5 mm; **d** Type D, kyphotic alignment at L4/5

**Fig. 2:**
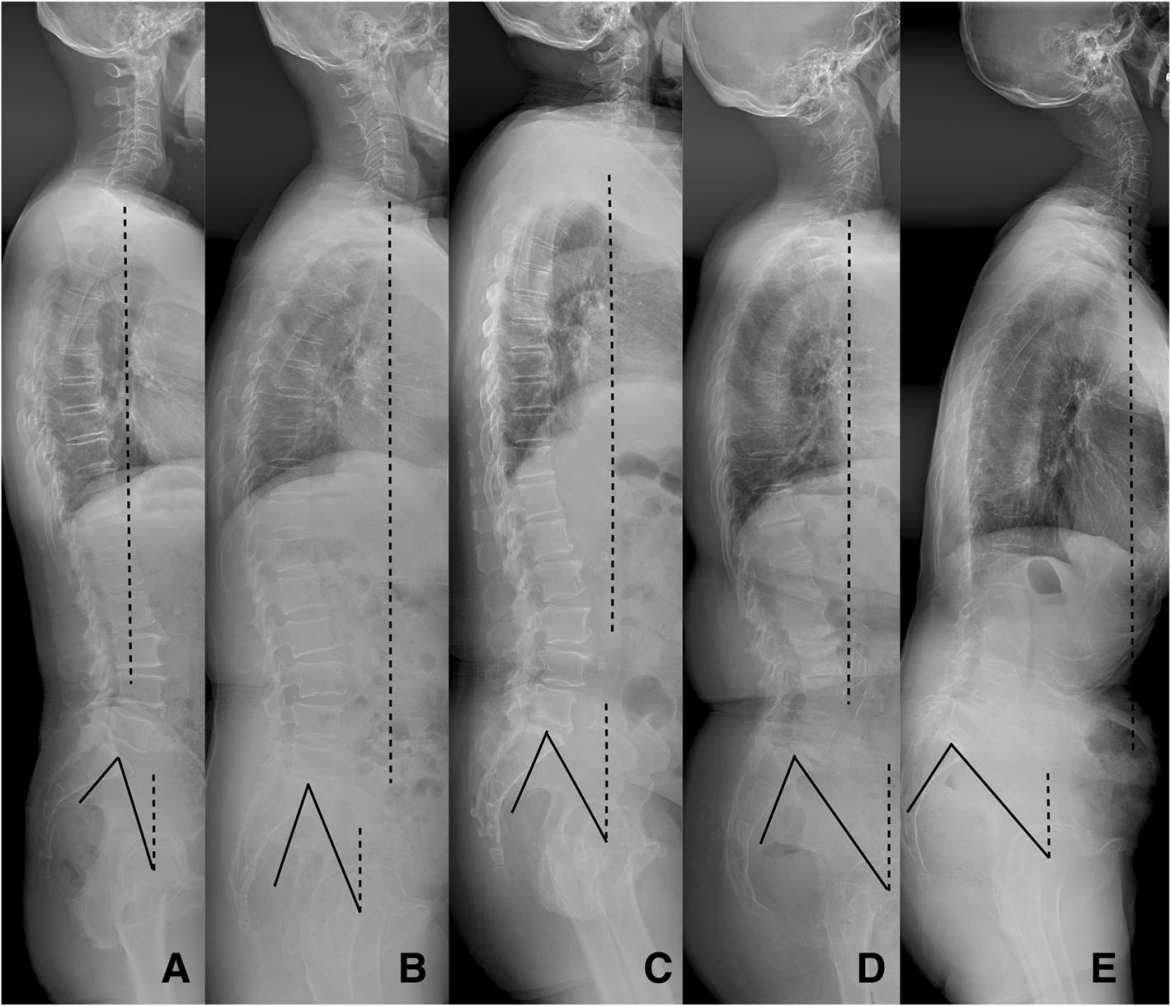
Diagram of the French classification. **a** Type 1, SVA (3.4 cm) < 4 cm, SL (16°) > 5°, LL (52°) > PI (55°)-10°; **b** Type 2, SVA (3.7 cm) < 4 cm, SL (5°) = 5°, LL (38°) > PI (44°)-10°; **c** Type 3, SVA (3.6 cm) < 4 cm, LL (41°) < PI (56°)-10°, PT (23°) < 25°; **d** Type 4, SVA (3.8 cm) < 4 cm, LL (38°) < PI (58°)-10°, PT (35°) > 25°; **e** Type 5, SVA (10.6 cm) > 4 cm

The CARDS classification [7] stratifies lumbar DS into four morphology subtypes based on three radiographic variables and one clinical variable (Fig. 1). The French classification system was derived from the adult spinal deformity classification system developed by Schwab et al. [9] (Fig. 2). Five types were defined based on sagittal balance, the relationship between LL and PI, and local segment lordosis as shown in Table 1.

**Table 1:** French classification system for lumbar DS

Definition	Type 1	Type 2	Type 3	Type 4	Type 5
SL	Preserved SL (> 5°)	Decreased SL (< 5°)	Decreased SL (< 5°)	Decreased SL (< 5°)	–
LL	Preserved LL (LL > PI-10°)	Preserved LL (LL > PI-10°)	Decreased LL (LL < PI-10°)	Decreased LL (LL < PI-10°)	–
PT	–	–	PT < 25°	PT > 25°	–
Sagittal balance	Balanced (SVA < 4 cm)	Balanced (SVA < 4 cm)	Balanced (SVA < 4 cm)	Balanced (SVA < 4 cm)	Sagittal unbalance (SVA > 4 cm)

Note: *SL* segmental lordosis, *LL* lumbar lordosis, *PI* pelvic incidence, *PT* pelvic tilt, *SVA* sagittal vertical axis

Four graders (2 orthopedic surgeons and 2 orthopedic fellows) rated all cases independently. Every grader received a 10-min tutorial on both classification systems before grading all patients. Three weeks later, all patients were regraded by each of the four graders after changing the order of the patients.

### Statistical analysis

For both classification systems, intra- and interobserver reliability were calculated and analyzed according to Kepler’s method [7]. The differences in age, gender, BMI, and preoperative and postoperative outcome scores were analyzed using the Kruskal–Wallis test between different subtypes in both French and CARDS classification systems. All statistical analyses were performed using SPSS 18.0 software with a significance level set at *P* < 0.05.

## Results

### Reliability analysis

Of the 146 patients included, 96 were female and 50 were male. The mean age was 62.4 ± 12.5 (42–81) years. The mean body mass index (BMI) was 26.74 ± 12.5 (20.41–33.06).

A total of 1168 grading times were made by four graders using the French classification system (146*4*2), including Type 1 (34.6%), Type 2 (17.2%), Type 3 (30.4%), Type 4 (9.4%), and Type 5 (8.4%). The consensus rate (interobserver agreement) reached for the French classification system was 83.6% (0.767–0.879). The κ value for interobserver reliability of all 146 patients was 0.693, representing substantial agreement. The κ value for intraobserver reliability patients was 0.743 (0.721–0.835), also representing substantial agreement (Table 2). Of the 1168 grading times made by four graders using the CARDS classification system, 3.4% were Type A1, 4.6% were Type A2, 11.2% were Type B1, 9.4% were Type B2, 37.8% were Type C1, 16.7% were Type C2, 12.4% were Type D1, and 4.5% were Type D2. The consensus rate (interobserver reliability) reached for the CARDS classification system was 89.6% (0.857–0.942). The κ value for interobserver reliability of all 146 patients was 0.837, representing perfect agreement. The κ value for intraobserver reliability was 0.869 (0.823–0.931), also representing perfect agreement (Table 3).

**Table 2:** Intraobserver reliability of the French classification of lumbar DS

	Cases in agreement between first and second observation			
	Grader 1	Grader 2	Grader 3	Grader 4
Type 1	40	44	39	41
Type 2	18	20	16	18
Type 3	30	37	37	35
Type 4	17	18	15	17
Type 5	10	10	14	12
Total	115	129	121	123
κ value	0.721	0.835	0.762	0.793

**Table 3:** Intraobserver reliability of the CARDS classification of lumbar DS

	Cases in agreement between first and second observation			
	Grader 1	Grader 2	Grader 3	Grader 4
Type A1	6	7	5	6
Type A2	8	8	9	7
Type B1	16	20	14	18
Type B2	12	13	17	16
Type C1	39	38	43	37
Type C2	21	24	20	23
Type D1	15	19	16	15
Type D2	8	10	9	6
Total	125	139	133	128
κ value	0.823	0.931	0.897	0.829

### Validity analysis

#### Demographics and baseline characteristics analysis

Patients of different CARDS types had comparable demographics as gender, age, and BMI (*P* > 0.05) (Table 4). All patients underwent lumbar fusion with instrumentation. Preoperative VAS (back) for Type D was higher than for other types (*P* = 0.036) (Table 4). Mean ODI and SF-36 scores of patients with different CARDS types had no significant differences (Table 4).

**Table 4:** Demographics and preoperative clinical scores of patients with different CARDS types

CARDS	Age	BMI	VAS back	VAS leg	ODI	SF-36
Type A	54.4 ± 9.3	29.14 ± 10.3	6.12 ± 4.3	5.89 ± 3.1	44.3 ± 17.7	43.9 ± 14.1
Type B	61.4 ± 11.6	30.74 ± 12.5	6.93 ± 3.7	6.26 ± 1.3	48.3 ± 23.1	42.1 ± 11.6
Type C	63.6 ± 13.1	25.32 ± 16.4	7.14 ± 2.1	6.68 ± 3.7	43.1 ± 21.3	40.3 ± 15.4
Type D	66.3 ± 16.5	26.29 ± 18.7	8.26 ± 1.7	7.45 ± 2.3	51.6 ± 19.5	38.5 ± 16.3
*P* value	0.431	0.519	0.036	0.136	0.482	0.548

With regard to the French classification, patients of different types also had no difference in demographics (*P* > 0.05) (Table 5). Mean back pain and leg pain for Type 5 were found to be significantly higher than those for all other French types (P1 = 0.017, P2 = 0.023) (Table 5). Patients of different French types had no differences in ODI scores or SF-36 scores (*P* > 0.05) (Table 5).

**Table 5:** Demographics and preoperative clinical scores of patients of different French types

French	Age	BMI	VAS back	VAS leg	ODI	SF-36
Type 1	52.1 ± 13.2	25.27 ± 9.9	5.71 ± 3.3	5.22 ± 1.8	40.3 ± 17.7	42.9 ± 14.1
Type 2	60.3 ± 14.7	30.14 ± 13.2	6.52 ± 2.5	5.78 ± 3.1	48.3 ± 23.1	41.1 ± 18.6
Type 3	59.4 ± 10.8	25.12 ± 11.6	7.11 ± 1.7	6.64 ± 1.6	43.1 ± 21.3	43.3 ± 21.4
Type 4	62.9 ± 16.1	26.12 ± 17.1	7.34 ± 0.8	6.61 ± 2.1	51.6 ± 19.5	39.5 ± 16.3
Type 5	68.1 ± 17.5	29.31 ± 16.4	8.41 ± 2.3	7.81 ± 3.2	52.7 ± 16.4	37.4 ± 17.6
*P* value	0.173	0.458	0.017*	0.023*	0.182	0.147

#### Postoperative outcome improvement analysis

One-year follow-up data was available for 146 of 158 patients (92.4%). All clinical outcome measures showed statistically significant improvement for the cohort as a whole and for each subtype in both classifications (*P* < 0.001). When comparing outcome improvements between different CARDS subtypes, Type D patients had greater improvements in back pain (*P* = 0.038) (Table 6). For the French classification, however, French type 5 showed the largest degree of improvement in all outcomes measures (*P* < 0.05) (Table 7).

**Table 6:** Mean Improvements in Outcome Scores According to CARDS Type

CARDS	VAS back	VAS leg	ODI	SF-36
Type A	4.14 ± 1.4	4.27 ± 1.8	21.4 ± 15.1	28.6 ± 16.1
Type B	4.92 ± 2.9	4.26 ± 1.3	20.7 ± 18.3	29.1 ± 12.4
Type C	4.46 ± 3.2	4.72 ± 2.4	23.1 ± 21.3	32.2 ± 16.2
Type D	6.47 ± 2.3	5.28 ± 2.1	32.4 ± 15.7	37.1 ± 18.3
*P* value	0.038*	0.331	0.287	0.348

**Table 7:** Mean Improvements in Outcome Scores According to French Type

French	VAS back	VAS leg	ODI	SF-36
Type 1	4.25 ± 1.6	3.82 ± 1.9	23.1 ± 16.3	26.7 ± 14.3
Type 2	5.12 ± 2.2	4.47 ± 1.4	22.4 ± 14.2	31.2 ± 13.6
Type 3	4.10 ± 1.4	3.98 ± 2.1	24.2 ± 18.1	33.6 ± 18.1
Type 4	4.57 ± 2.2	4.88 ± 1.5	29.6 ± 16.3	32.6 ± 11.3
Type 5	7.02 ± 2.4	6.47 ± 1.7	33.2 ± 17.1	37.8 ± 15.2
*P* value	0.038*	0.041	0.0824	0.120

## Discussion

Lumbar DS is a common spinal pathology, with anterior displacement of one vertebral body over another caused by degeneration of lumbar disc and facet joints [10]. When conservative treatment fails, surgery is often advocated [11, 12]. A variety of surgical approaches have been advocated, such as decompression alone, fusion with or without instrumentation, and decompression with dynamic stabilization. However, there is no consensus among spinal surgeons regarding optimal surgical treatments for patients with lumbar DS [13, 14]. The variability of radiographic features suggests that lumbar DS is a heterogeneous disease and requires a specific grading system [15].

The Meyerding classification, though widely used for describing the degree of spondylolisthesis, is less useful for lumbar DS since almost all cases of lumbar DS would fall into grade I or II category [16]. Also, the Meyerding classification does not take other morphologic parameters such as segmental kyphosis or disc height into consideration, which are related to clinical outcomes [17].

Recently, the CARDS classification was proposed, which highlighted the role of disc height and segmental kyphosis. This classification is based on clinical symptoms and three radiographic parameters: disc height, anterior translation, and the presence of segmental kyphosis. Type A indicates severe degeneration of intervertebral disc but the stability may be maintained [18]. The difference between Type B and C is the distance of translation. Type D means segmental kyphosis, which indicates disc degeneration and back pain. Although simple and easy to understand and use in clinical practice, the CARDS classification does not include spinopelvic parameters. Another newly proposed classification, The French classification, takes comprehensive consideration of disc height and spinopelvic compensation. This classification has therapeutic implications according to the authors, as severity increases from Type 1 to Type 5.

In order for any new classification system to be successfully accepted, it must be shown to be reliable and reproducible to ensure consistent application of the system. Additionally, the system should be proven clinically relevant to provide further decision-making. To the best of our knowledge, this is the first study to compare the reliability and validity between the CARDS and French classifications.

Most lumbar DS patients in this study were subdivided into Types B and C according to the CARDS classification, with partial preservation of disc height and no segmental kyphosis. The κ value for interobserver reliability was 0.837, representing perfect agreement. The κ value for intraobserver reliability was 0.869, also representing perfect agreement. Our results were consistent with the findings of Kepler et al. [7] and Sobol et al. [19], which showed that the CARDS classification is reliable and reproducible. Few studies have determined the reliability of the French classification. We found that the interobserver κ value for the French classification was 0.693 and the intraobserver κ value was 0.743, both representing substantial agreement. According to this study, the CARDS classification had better reliability. The main reasons may be the easier grading system and fewer parameters measured in the CARDS system.

In the CARDS classification system, Types A and D are easy to determine and the difference between Types B and C is the distance of translation. Thus, only one parameter needs to be measured. The French classification system, however, is based on sagittal balance, relationship between LL and PI, and local segment lordosis, requiring high quality radiographs and accurate measurement of many spinopelvic parameters. The bias arising from measurements of parameters may contribute to the lower reliability of the French system. Nevertheless, the French classification system has substantial agreement for both inter and intraobserver reliabilities.

This study also confirmed previous findings that CARDS Type D patients have higher preoperative back pain scores compared with non-Type D patients [17]. Chen et al. [19] demonstrated that Type D spondylolisthesis was associated with dynamic instability at the involved segment. One possible theory is that Type D patients have insufficient anterior column support, which severely impairs their ability to resist anterior shear forces. This theory may also explain why CARDS Type D patients benefit more from surgery with greater improvement in back pain, as shown in this study. In addition, many studies have suggested that interbody fusion is reasonable in patients with CARDS Type D classification [20–22]. Our study validates the clinical utility of the CARDS classification system and that Type D spondylolisthesis is a rational prognostic indicator for interbody fusion.

This French classification system has therapeutic implications according to the authors, as severity increases from Type 1 to Type 5. Mean back pain and leg pain for Type 5 were significantly higher than those for all other French types. The altered overall balance in Type 5 patients may lead to the mechanical back pain and a deterioration of spinal stenosis, which can be reflected by higher back pain and leg pain. The findings of this study also suggested that Type 5 patients may be prone to achieve greater improvement in back and leg pain when compared with other subtypes. According to Gille et al. [8], different surgical strategies were suggested for different French types: simple posterior fusion for Type 1; restoration of SL for Type 2; correction of LL for Type 3; mandatory restoration of LL for Type 4; and correction of sagittal deformity for Type 5. However, those hypotheses require further validation in larger studies.

This study had several limitations. First, this was a retrospective study with a small sample size and relatively short follow-up time. Second, this graders included two orthopedic spinal surgeons and two orthopedic spinal fellows which is a confounding variable. Third, 3 weeks’ interval may be not enough between gradings and the tutorial may increase interobserver agreement. Forth, statistical significance in this study does not guarantee clinical significance. The minimum clinically important difference (MCID) represents the smallest improvement considered worthwhile by a patient, which may be better than statistical significance in this study. However, only patient reported outcomes as VAS, ODI, and SF 36 were included and there was no external criterion used as anchor. Because of the lack of proper anchor, we were unable to get a reliable MCID. All these factors may cause bias in this study. Finally, our results may not be entirely representative of all DS patients since the cohort was obtained from a single institution.

## Conclusion

In conclusion, both CARDS and French classification systems have acceptable reliability. The CARDS system is easier to utilize and has better reliability. CARDS Type D patients tend to have worse preoperative back pain and greater improvement after surgery. The French system highlights the role of sagittal alignment and balance, and may provide more information for decision-making. French Type 5 patients have higher preoperative back pain and greater improvement after surgery. Further studies are needed to confirm our results and clarify the prognostic value of these two systems.

## Data Availability

The datasets used and/or analyzed during the current study are stored in our hospital and are available from the corresponding author on reasonable resquest.

## Abbreviations

BMI: Body mass index
CARDS: The Clinical and Radiographic Degenerative Spondylolisthesis
DS: Degenerative Spondylolisthesis
IDH: Intervertebral disc height
LL: Lumbar lordosis
NASS: The North American Spine Society
ODI: Oswestry Disability Index
PI: Pelvic incidence
PT: Pelvic tilt
SF-36: 36-Item Short Form Health Survey
SL: Segmental lordosis
SVA: Sagittal vertical axis
VAS: Visual analog scale
